# Supplementary material for: The genetic basis of natural variation in a phoretic behavior
Source: Nat Commun. 2017 Aug 17;8:273. doi: 10.1038/s41467-017-00386-x (PMC5561207; doi:10.1038/s41467-017-00386-x)
Supplement: Supplementary file 1 — Supplementary Information [file 41467_2017_386_MOESM1_ESM.pdf]

File Name: Supplementary Information
Description: Supplementary Figures and Supplementary Tables.

File Name: Supplementary Movie 1
Description: ***Caenorhabditis* dauer larva hitchhiking on a small beetle** Dauer larva of *C. latens* nictating on the back of a fast-walking beetle on the surface of a rotten apple.

File Name: Supplementary Movie 2
Description: **Nictating *Caenorhabditis* dauer larva** Dauer larva of *C. latens* nictating on the tip of a rotten apple.

File Name: Supplementary Data 1
Description: **Natural variation of nictation behavior** Nictation fractions of 12 divergent *C. elegans* wild isolates are included.

File Name: Supplementary Data 2
Description: **Nictation variation among RIAIL strains** Nictation fractions of 186 recombinant inbred advanced intercross lines (RIAILs) are included.

File Name: Supplementary Data 3
Description: **NIL-based fine mapping of *nict-1* QTL** Nictation fractions of near isogenic lines (NILs) are included.

File Name: Supplementary Data 4
Description: **Genomic variants in *nict-1* QTL** Genomic features and variants of NILnarrowed 73 kb *nict-1* QTL are included.

File Name: Supplementary Data 5
Description: **Expression analysis for *nict-1* QTL** Expression levels of genes in the NILnarrowed 73 kb *nict-1* QTL are included.

File Name: Supplementary Data 6
Description: **Candidate gene assay** Nictation fractions of strains with deletion mutations in genes in the NIL-narrowed 73 kb *nict-1* QTL are included.

File Name: Supplementary Data 7
Description: **Brood size assay** Brood sizes of the parental and NIL strains are included.

File Name: Supplementary Data 8
Description: **Transmission competition assay** Isopod transmission competition assay allele frequencies are included.

File Name: Peer Review File

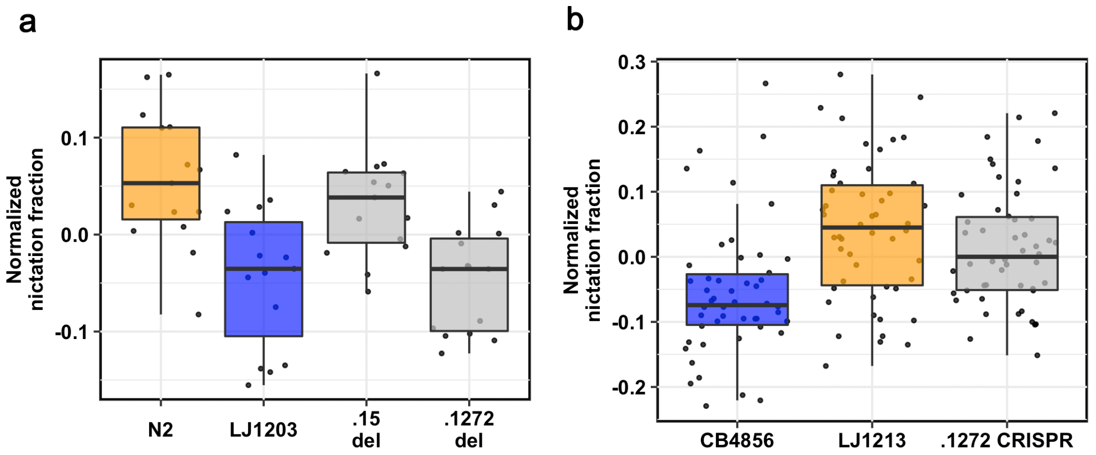

Supplementary Figure 1. The nictation difference is not regulated by protein-coding genes in *nict-1* QTL. Tukey box plots of normalized nictation percent of are shown with data points plotted behind. The horizontal line in the box is the median, and the box denotes the 25th to 75th quantiles of the data. The vertical line represents the 1.5 inter-quartile range. (**a**) N2 (orange), LJ1203 (*nict-1* CB4856>N2, blue), FX1161 *Y105C5A.15(tm1161)* and FX6979 *Y105C5A.1272(tm6979)* were scored in 15 biological replicates. (**b**) CB4856(blue), LJ1213 (*nict-1* N2>CB4856, orange) and LJ1216 (*nict-1* N2>CB4856); *Y105C5A.1272*(*snu33*) were scored in 50 biological replicates.

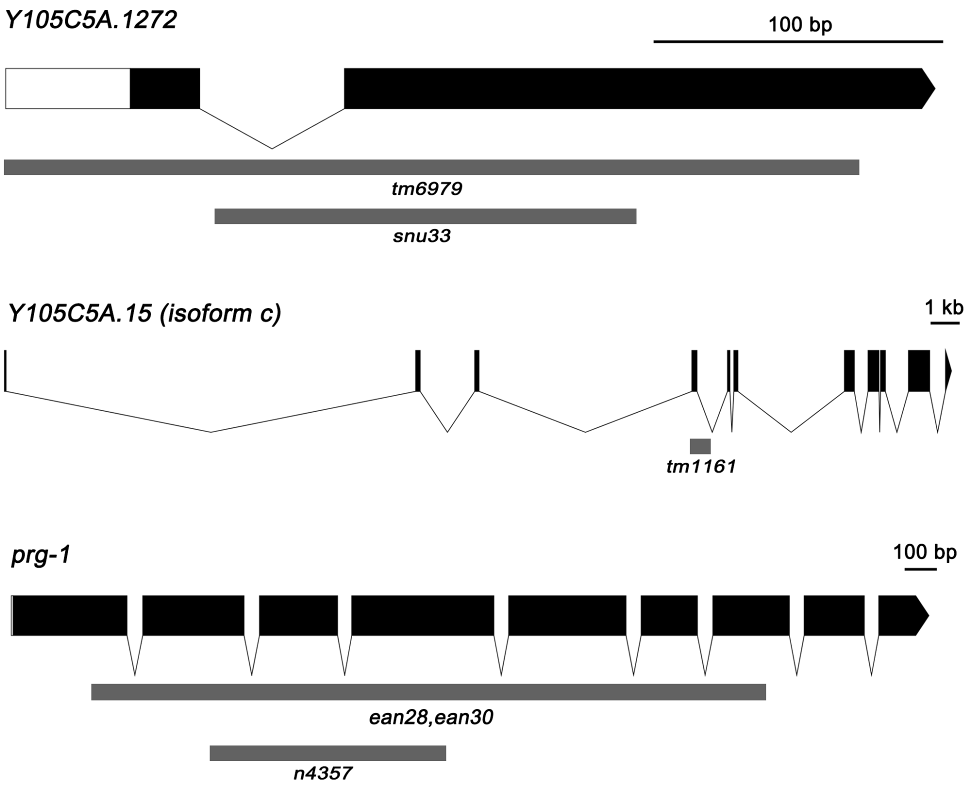

Supplementary Figure 2. Deletion mutations in candidate genes and the *prg-1* gene. The locations and sizes of the deletions are marked by gray rectangles.

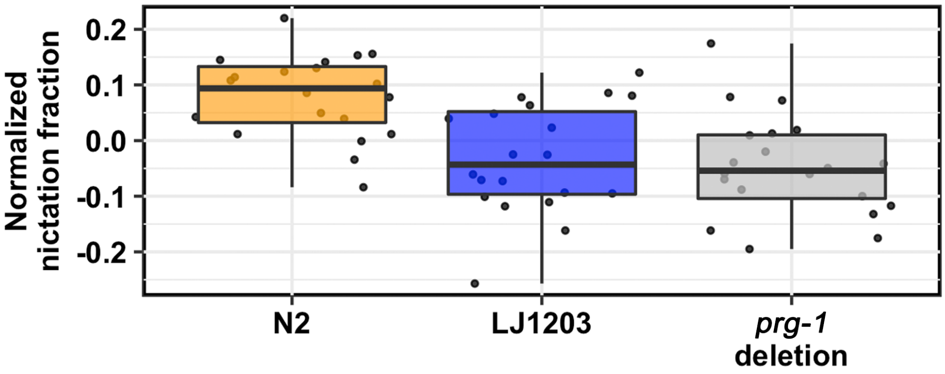

Supplementary Figure 3. An independent *prg-1* mutation allele causes a decrease in nictation frequency. Tukey box plots of normalized nictation percent of N2 (orange), LJ1203 (*nict-1* CB4856>N2, blue), and ECA286 *prg-1(n4357)* are shown with data points plotted behind. The horizontal line in the box is the median, and the box denotes the 25th to 75th quantiles of the data. The vertical line represents the 1.5 inter-quartile range. All strains were scored in 20 biological replicates.

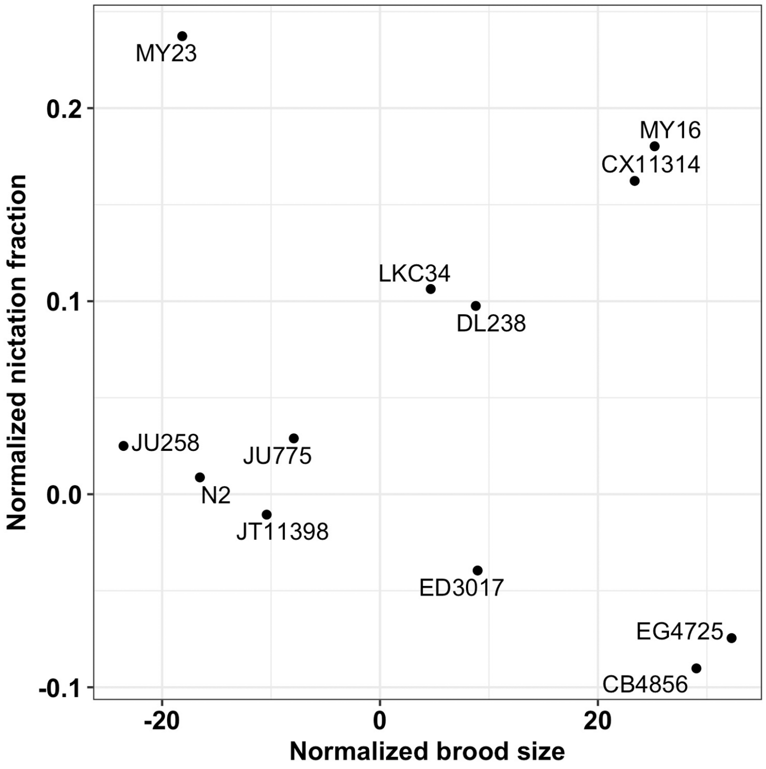

Supplementary Figure 4. The correlation between nictation and offspring production of wild isolates were analyzed. A scatter plot of normalized nictation fractions and normalized brood sizes of 12 *C. elegans* wild isolates are shown.

Strain	Left breakpoint marker	Right breakpoint marker
LJ1205	15,363,068 (haw64325)<br>15,399,910 (haw64411)	15,721,883(haw64913)<br>15,731,721(CE4-211)
LJ1206	15,363,068 (haw64325)<br>15,399,910 (haw64411)	15,570,359 (haw64768)<br>15632637 (CE4-210)
LJ1207	15,731,721 (CE4-211)<br>15,769,804 (haw64984)	15,874,012 (haw65181)<br>15,907,604 (haw65266)
LJ1209	15,363,068 (haw64325)<br>15,399,910 (haw64411)	15,731,721 (CE4-211)<br>15,769,804 (haw64984)
LJ1210	15,363,068 (haw64325)<br>15,399,910 (haw64411)	15,787,240 (haw65037)<br>15,812,722 (haw65069)
LJ1211	15,714,544 (cewivar00695185)<br>15,718,040 (cewivar00695189)	15,874,012 (haw65181)<br>15,907,604 (haw65266)
LJ1212	15,363,068 (haw64325)<br>15,399,910 (haw64411)	15,645,536 (haw64838)<br>15,646,308 (haw64840)
LJ1213	15,644,563 (cewivar00215554)<br>15,645,536 (haw64838)	15,787,240 (haw65037)<br>15,812,722 (haw65069)
LJ1215	15,589,708 (haw64785)<br>15,641,444 (haw64835)	15,731,721 (CE4-211)<br>15,769,804 (haw64984)

Supplementary Table 1. Breakpoint markers for the generation of near isogenic lines (NILs)

Position	Forward primer	Reverse primer
15,363,068	CCGTACCTCGGAAGTTGAAA	AAGAGCCATAACACATGAGCTG
15,399,910	ATGGTGGTTTTCAAGAGTTTTCA	GCTGGAATCAATTAAGAAATTACAAA
15,570,359	TGACCGAAAATGAAAAATTCG	TCCCTTACTCACCCTGTCACTT
15,570,612	TGACCGAAAATGAAAAATTCG	TCCCTTACTCACCCTGTCACTT
15,589,708	TGCGGTAAAGTCGAATTTTTA	AAAAGCAATGCTGATGATGC
15,632,637	ATCGGATGTTTCAGCTTGTGG	ATACGGAGGATTTTGCATGTCC
15,641,444	AGGCGAATGACTCATGCTTT	AGCCAAAATGCCCACTACAC
15,644,563	CATTGGCTGGTCCCACTC	AAATGTTGGCCTTGTTGAAAA
15,645,536	TTTTCAACAAGGCCAACATTT	AATGATCCGCAACTTTTTCG
15,646,308	TGGTATTGTAGAAAATAAAAAGTTCCA	AAAGGGTGAGTACGCAATGG
15,696,457	TCGAAAATTCTAAAACCGAACT	AAAACGGAAGGACATGTTGTG
15,714,544	GCCCCATCGAAACAGTAGAA	CGTGGGAAAACACACACTGA
15,718,040	CAACCAATATCACGCTGTCG	CGCCACGCAGTTTCTTATTT
15,721,883	TTCATGATGTTCATTTATTTTTGGA	TTTCTGTCACAGCCAAGTTCA
15,731,721	ACCATTCTTTCAGATTGTTCCT	CAATACTGATGAAAACTGTTTGA
15,769,804	TCACCACTGGTTTTCACTTCA	AAAAAGTGTGAATTCGTTTTGGA
15,787,240	TTTCAAATGTGCTTTGCTAATGA	AAATTTTGAAACAGGAACAAAAA
15,812,722	AAAATCAGAATTTGAACTGTTTTGG	CATCGAAAATTTACAGTTTCACATTA
15,874,012	GCTCCGCTGAGCACTAAAGA	TGAAGGATGCGATACTCTCTCTC
15,907,604	AGCATTTCTCACGCGCAG	TTTTGCTGTTTCGAATCAAACA
16,003,539	ATCTTTGAAGCAGCGGACAT	TTGAAAGTTTACAAGTCAAGCACA

Supplementary Table 2. Primer sequences for the markers used to genotype the NILs
